# Management of Difficult Airway With Laryngeal Mask in a Child With Mucopolysaccharidosis and Mitral Regurgitation: A Case Report

**DOI:** 10.5812/cardiovascmed.17456

**Published:** 2014-04-01

**Authors:** Mohsen Ziyaeifard, Rasoul Azarfarin, Rasoul Ferasatkish, Majid Dashti

**Affiliations:** 1 Rajaie Cardiovascular Medical and Research Center, Iran University of Medical Sciences, Tehran, IR Iran

**Keywords:** Mucopolysaccharidosis, Airway Management, Anesthesia, Laryngeal Masks

## Abstract

**Introduction:**

Mucopolysaccharidoses (MPSs) are a group of heredity storage diseases, transmitted in an autosomal recessive manner, associated with the accumulation of glycosaminoglycans (GAGs) in various tissues and organs. The concerned patients have multiple concomitant hereditary anomalies. Considering the craniofacial abnormality in these patients, airway management may be difficult for anesthesiologists. In these patients, preanesthetic assessment is necessary and performed with the accurate assessment of airways, consisting of the physical exam and radiography, MRI or CT of head and neck. An anesthesiologist should set up a “difficult intubation set” with a flexible fiber-optic bronchoscope and also, it may be necessary to discuss with an ear-nose and throat (ENT) specialist if required, for unpredicted emergency situations.

**Case Presentation:**

In this case-report we presented a 2-year-old boy with known MPSs with psychomotor retardation, bilateral corneal opacities, impaired hearing and vision, inguinal hernia, severe mitral regurgitation, micrognathia, coarse facial feature, stiff and short neck and restricted mouth opening. He scheduled for left inguinal hernia repair surgery.

**Discussion:**

The patient’s difficult airway was managed successfully and the anesthesia of his surgical procedure had an uneventful course.

## 1. Introduction

One of the most important responsibilities of an anesthesiologist is airway management and therefore it is essential to perform a complete airway evaluation before initiation of anesthesia (1). Difficult airway management is always an important problem. Ventilation failure and hypoxia are the main reasons of neurological insufficiency and may lead to death in patients with difficult airway (2). The mucopolysaccharidoses (MPSs) represent a group of hereditary storage diseases that are transmitted in an autosomal recessive manner. They are associated with the accumulation of glycosaminoglycans (GAGs) in different tissues and organs. These abnormalities cause progressive organ dysfunction and multi-systemic involvement. The incidence of this disease is about 1:50000 (3). The diagnosis of such patients is based on enzyme deficiencies in the serum and elevated mucopolysaccharide levels in the urine (4). The clinical manifestations of MPSs consist of ear, nose and throat anomalies, coarse face, skeletal deformity, cervical unsteadiness, kyphoscoliosis, growth destruction, hepatosplenomegaly, visual and hearing impairment, contractures in joints, hernias and cardiac lesions (valve involvement, myocarditis and endocarditis) and also respiratory disease (5). Most MPS patients require anesthesia for numerous surgical interventions. These patients can have an amplified anesthetic risk secondary to a difficult airway (6). The surgery, in these patients, is associated with an elevated mortality rate. The anesthetic complications occurring during the surgical procedures are associated with airway obstruction, due to difficulty in ventilation and oxygenation. In these patients, due to the short neck, even the surgical tracheostomy can be difficult (7, 8). In this case-report, we present a two-year-old boy with MPS, mental retardation and bilateral corneal opacities, that referred with inguinal hernia and severe mitral regurgitation, and was proposed for hernia repair surgery.

## 2. Case Presentation

**Figure 1.**
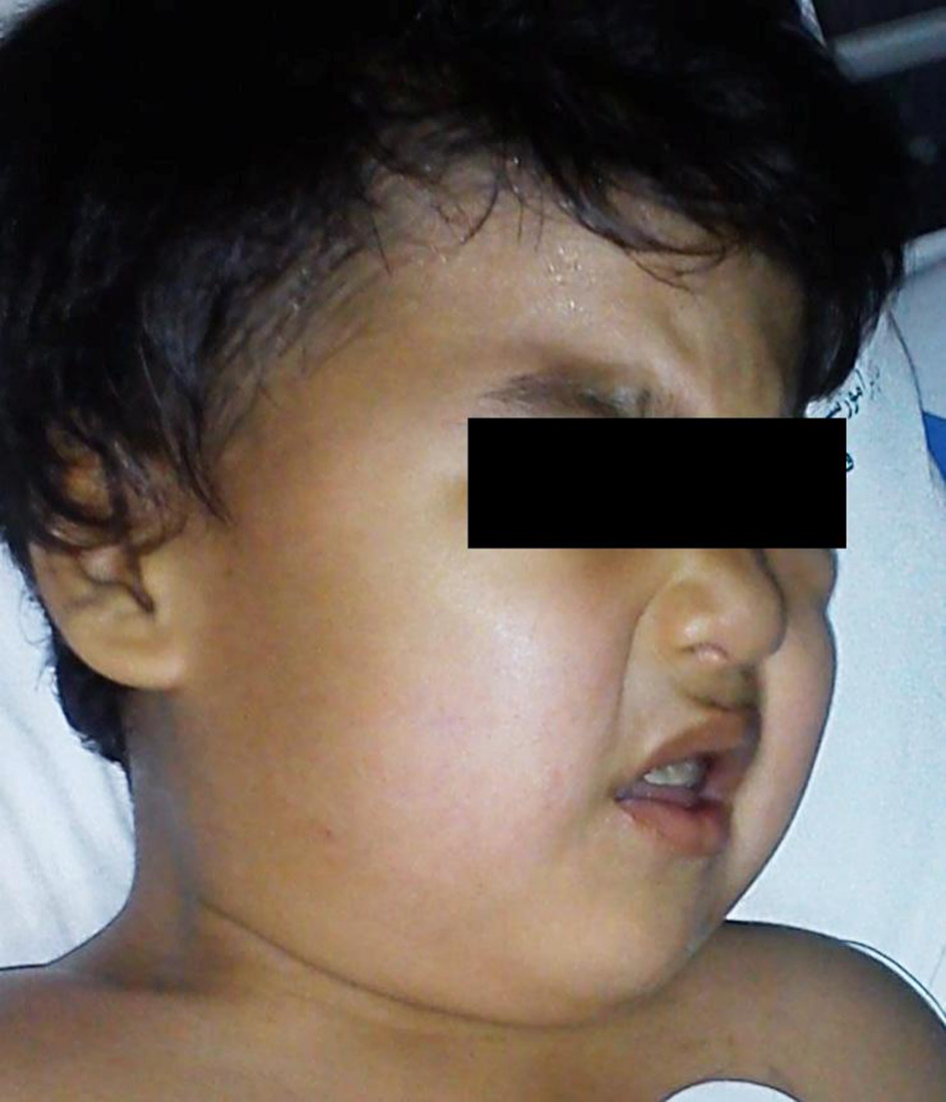
Coarse Facial Features in the Patient

**Figure 2.**
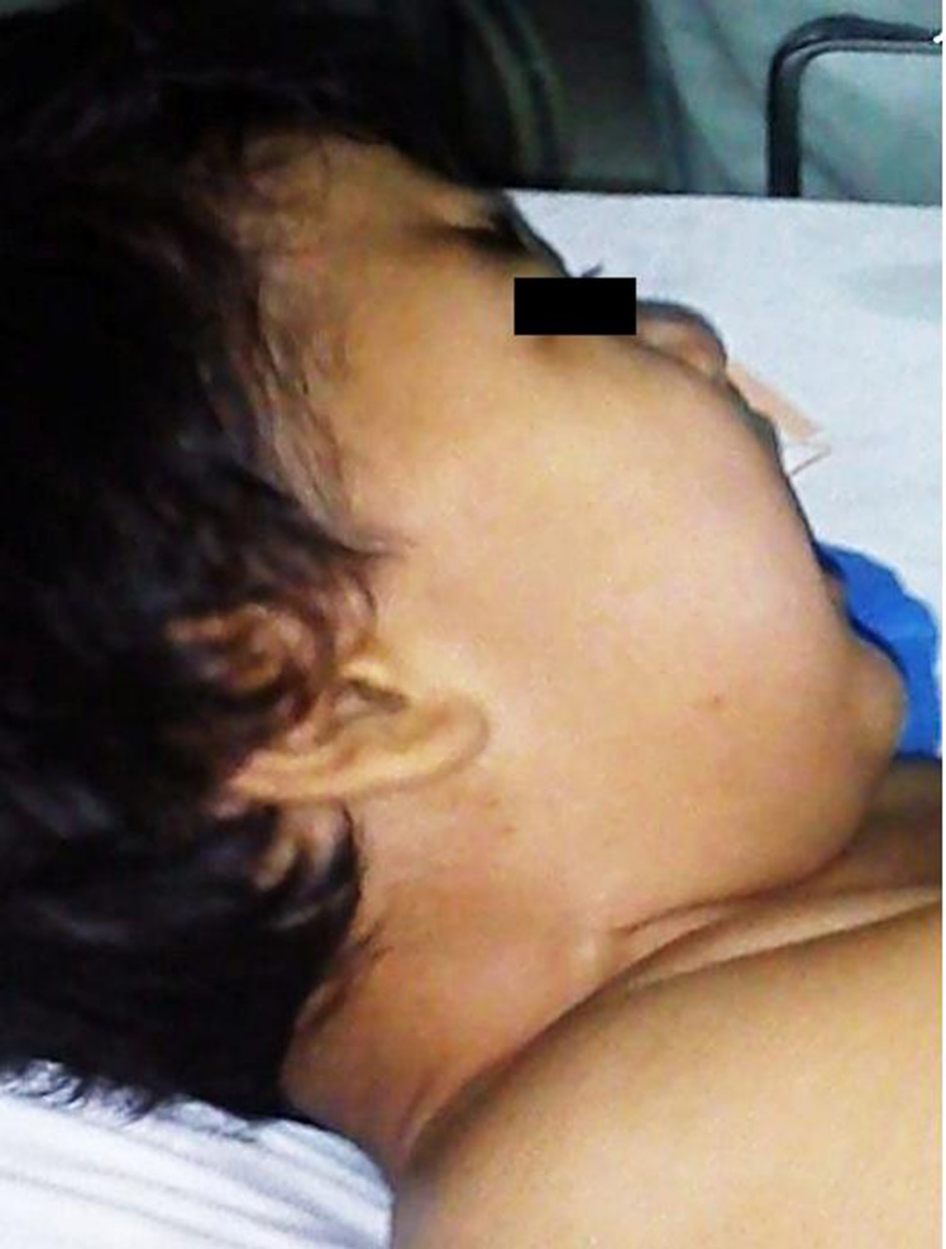
Stiff and Short Neck in the Patient

**Figure 3.**
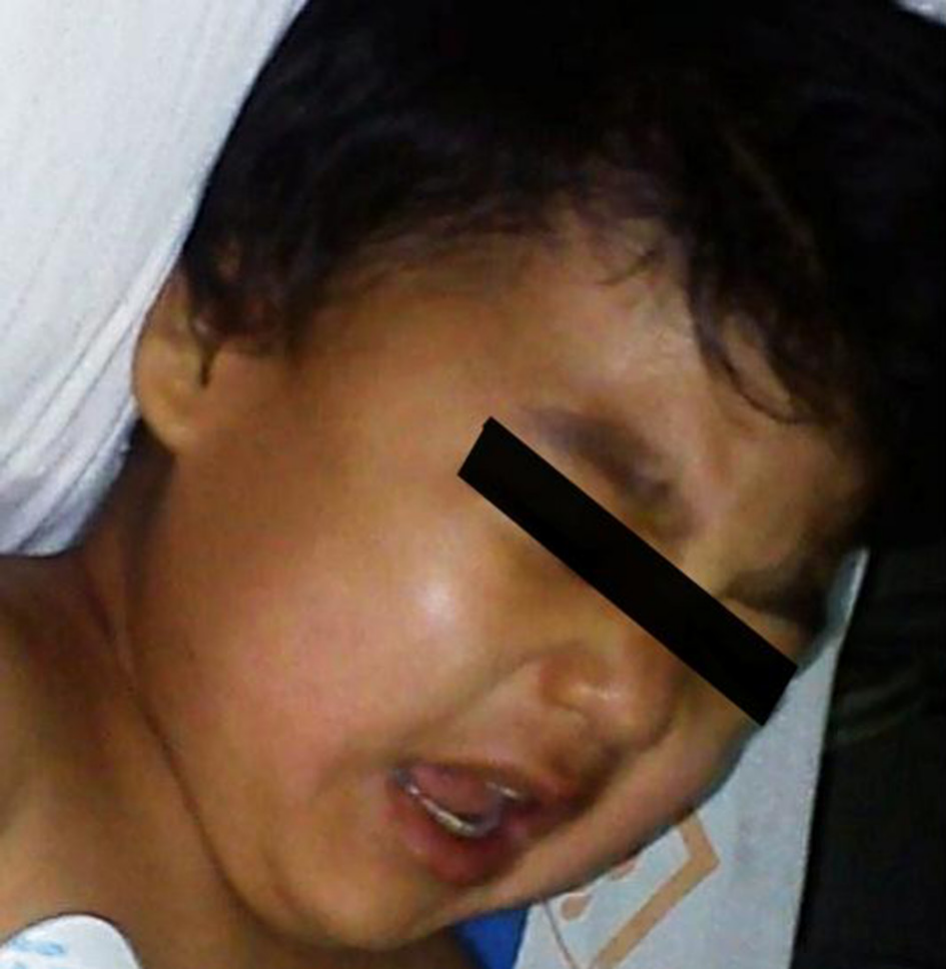
Restricted Mouth Opening in the Patient

A 2-year-old boy with a known history of MPS was scheduled for elective hernia repair surgery under general anesthesia. The patient’s weight was 20 kg and his height was 100 cm. The physical exam revealed psychomotor retardation, bilateral corneal opacities, impaired hearing and vision, inguinal hernia, micrognathia, coarse facial features (Figure 1), stiff and short neck (Figure 2), restricted mouth opening (Figure 3) and macroglossia, that orientated the medical team towards the possibility of difficult airway (Mallampatti score = 3). Therefore, we used a “difficult intubation set”, including a flexible fiber-optic bronchoscope. During the echocardiographic exam, the patient presented severe mitral regurgitation.

In the preoperative visit, the anesthesiologist ordered oral diphenhydramine 1 mg/kg as premedication 2 hours before surgery. The patient was monitored in the operating room with peripheral oxygen saturation (SPO_2_), electrocardiogram, and non-invasive blood pressure measurement. At first step, the patient was sedated by face mask inhalatory sevoflurane 3% in 100% oxygen. After, a peripheral IV cannula (20 G) was inserted and intravenous infusion of Ringer’s solution was initiated to a total amount of 8 mL/kg. The anesthesia induction was accomplished by midazolam 0.05 mg/kg, fentanyl 2 µ/kg and cisatracurium 0.15 mg/kg. The lungs were easily ventilated by face mask without any problem. The first attempt of tracheal intubation has been unsuccessfully performed by the anesthesia resident (laryngoscopy grade was 3). At the next effort, anesthesia attendant inserted the size 2 laryngeal mask airway (LMA) after cuff lubrication into the patient’s pharynx. The LMA cuff was inflated with a suitable volume of air (10 mL). Afterwards, we checked the correct position of LMA by visualization of air movement and auscultation of the lungs. We also used capnography to confirm the establishment of adequate ventilation. Anesthesia was maintained by sevoflurane 2% - 3%, fentanyl 1 µg/kg/hour and bolus doses of 0.05 mg/kg cisatracurium. Hernia was repaired and the operation was completed after about 1 hour. After completion of the operation, administration of anesthetic drugs was discontinued. Once the patient was awake and resumed spontaneous breathing, we administered atropine 0.02 mg/kg and neostigmine 0.04 mg/kg to reverse the effects of the neuromuscular blocking agent. The LMA was extracted and the patient was transferred to the ward after being fully awaken and responding positively to commands. Finally, this patient was discharged from the hospital 2 days later without any problem.

## 3. Discussion

The core problem in MPS is the accumulation of GAGs in various organ systems. The prominent manifestations of these disease groups are coarse facial features, dysostosis multiplex, hepatosplenomegaly, hernias, and recurrent respiratory infections (9). The high incidence of airway obstruction and restrictive pulmonary disease in the setting of cardiovascular manifestations pose a high anesthetic risk for these patients. The typical anesthetic complication includes airway obstruction after tracheal intubation or extubation, which may require emergency tracheostomy (10). Careful assessment of anesthetic risk factors should be made before the process, relating the assessment of airways and cardio-pulmonary and cervical spine troubles (6, 11). Cardiac problems are common in MPS. Therefore, it is essential to evaluate the cardiac risk before any attempt of general anesthesia. Heart valve disease is the most frequent cardiac finding in MPS, aortic and mitral regurgitation accounting for the clinical and echocardiographic characteristics encountered (12). In general, surgery in MPS patients is related with a high mortality rate. The most severe anesthetic problems occurring during surgery in MPS patients are coupled with airway obstruction, with additional difficulty in ventilation and oxygenation, resulting in important cardiovascular compromise (13). In the patients with unstable neck, cervical movement during intubation can lead to spinal cord damage and paralysis (4). Therefore, widespread imaging studies and discussions with related specialties, such as ear-nose-throat (ENT) or neurosurgery professionals, may be necessary (14).

Airway management has, at all times, been of crucial importance for the physicians of all specialties (15). Currently, an emphasis is placed on the education in airway management (16) by using proper instruments (17), especially in pediatric patients undergoing cardiac procedures. Several etiologies of airway deformity are congenital laryngeal stenosis, cleft palate, Down’s syndrome, Klippel-Feil syndrome, epiglottitis (18), lingual tonsillitis, abscess, laryngeal trauma, angioedema, thalassemia major, Turner, Stevens-Johnson, Marfan and Cornelia de Lange syndromes, and finally, MPS (19). Spontaneous ventilation methods using oxygen and a high-concentration volatile anesthetic have been frequently used. However, they require a skilled team to patent the airway if airway obstruction is encountered. Insertion of a LMA will often improve ventilation. Myorelaxants are best mislaid until endotracheal intubation has been reached and the airway is protected. The most suitable uncuffed endotracheal tube is frequently two or three sizes lesser than predicted for age (20). Preparation for extubation should consist of using intraoperative corticosteroids, complete reversal of the muscle relaxant and assignment of a nasopharyngeal airway to decrease upper airway obstruction after extubation. Anesthesia for minor surgical intervention can be done either by a face mask with a spontaneous breathing technique or an LMA. Intubation is not always necessary for minor surgeries, therefore avoiding intubation and extubation difficulties (21). In this case-report we present a 2-year-old boy with MPS, mental retardation and bilateral corneal opacities, that referred with inguinal hernia and severe mitral regurgitation and was proposed for hernia repair surgery. Difficulty in airway management of this patient was mainly due to craniofacial and skeletal malformations, short and stiff neck, the restricted opening of the mouth (Mallampatti score = 3), which consequently determined the operating team to prepare a “difficult intubation set” including a flexible fiberscope. After induction of anesthesia, the lungs were ventilated with a face mask without difficulty. Tracheal intubation was unsuccessful at first attempt. During the next effort, anesthesia attend inserted the size two LMA after cuff lubrication into the patient’s pharynx. In relation to the short operation time, we primarily decided to place LMA to secure the patient’s airway, therefore becoming unnecessary to perform fiber-optic bronchoscopy (FOB) at first step. However, when facing difficulty in the placement of LMA, the next step should be to use FOB. 

In pediatric patients with difficult airway, intubation with the aid of flexible fiberscope and local anesthetics coupled with mild sedation represents the method of choice for tracheal intubation in the setting of limited mouth opening. The smallest size of the fiber-optic bronchoscope is 1.8 mm, which can pass through a 2.5 mm ID endotracheal tube. This technique allows for simultaneous oxygen delivery directly into the trachea, as well as airway suctioning (22). Another option for these patients is stylet-guided endotracheal intubation after anesthesia induction. Introducing a Fastrach laryngeal mask through a LMA has been suggested as a substitute procedure for tracheal intubation in these patients (23). Tracheostomy must be performed when other alternative measures are unsuccessful (20). In conclusion, in patients with craniofacial anomalies such as in MPS, the anesthetic airway management is often challenging and exact preoperative evaluation alongside preparation of a ”difficult intubation set” can potentially reduce airway management complications.

## References

[1] Cattano D, Killoran PV, Iannucci D, Maddukuri V, Altamirano AV, Sridhar S (2013). Anticipation of the difficult airway: preoperative airway assessment, an educational and quality improvement tool.. Br J Anaesth..

[2] Rodrigues AJ, Scordamaglio PR, Palomino AM, de Oliveira EQ, Jacomelli M, Figueiredo VR (2013). Difficult airway intubation with flexible bronchoscope.. Rev Bras Anestesiol..

[3] Walker R, Belani KG, Braunlin EA, Bruce IA, Hack H, Harmatz PR (2013). Anaesthesia and airway management in mucopolysaccharidosis.. J Inherit Metab Dis..

[4] Valayannopoulos V, Nicely H, Harmatz P, Turbeville S (2010). Mucopolysaccharidosis VI.. Orphanet J Rare Dis..

[5] Sayilgan C, Yuceyar L, Akbas S, Erolcay H (2012). Anesthesia in a child with Maroteaux-Lamy syndrome undergoing mitral valve replacement.. Clinics (Sao Paulo)..

[6] Kamin W (2008). Diagnosis and management of respiratory involvement in Hunter syndrome.. Acta Paediatr Suppl..

[7] Chan YL, Lin SP, Man TT, Cheng CR (2001). Clinical experience in anesthetic management for children with mucopolysaccharidoses: Report of ten cases.. Acta Paediatr Taiwan..

[8] Suh SH, Okutani R, Nakasuji M, Nakata K (2010). Anesthesia in a patient with mucopolysaccharidosis type VI (Maroteaux-Lamy syndrome).. J Anesth..

[9] Herrick IA, Rhine EJ (1988). The mucopolysaccharidoses and anaesthesia: a report of clinical experience.. Can J Anaesth..

[10] Muhlebach MS, Wooten W, Muenzer J (2011). Respiratory manifestations in mucopolysaccharidoses.. Paediatr Respir Rev..

[11] Wold SM, Derkay CS, Darrow DH, Proud V (2010). Role of the pediatric otolaryngologist in diagnosis and management of children with mucopolysaccharidoses.. Int J Pediatr Otorhinolaryngol..

[12] Wippermann CF, Beck M, Schranz D, Huth R, Michel-Behnke I, Jungst BK (1995). Mitral and aortic regurgitation in 84 patients with mucopolysaccharidoses.. Eur J Pediatr..

[13] John A, Fagondes S, Schwartz I, Azevedo AC, Barrios P, Dalcin P (2011). Sleep abnormalities in untreated patients with mucopolysaccharidosis type VI.. Am J Med Genet A..

[14] Taguchi S, Kusunoki S, Fukuda H, Hamada H, Kawamoto M (2009). [Difficult tracheal intubation using airway scope in a pediatric patient with Hunter syndrome].. Masui..

[15] Golzari SE, Khan ZH, Ghabili K, Hosseinzadeh H, Soleimanpour H, Azarfarin R (2013). Contributions of Medieval Islamic physicians to the history of tracheostomy.. Anesth Analg..

[16] Soleimanpour H, Gholipouri C, Panahi JR, Afhami MR, Ghafouri RR, Golzari SE (2011). Role of anesthesiology curriculum in improving bag-mask ventilation and intubation success rates of emergency medicine residents: a prospective descriptive study.. BMC Emerg Med..

[17] Ziyaeifard M, Azarfarin R, Massoumi G (2012). A comparison of intraocular pressure and hemodynamic responses to insertion of laryngeal mask airway or endotracheal tube using anesthesia with propofol and remifentanil in cataract surgery.. J Res Med Sci..

[18] Mofateh M, Ziaei fard M, Makhmalbaf G, nabei M (2008). A case report of Post-Varicella Epiglottitis.. J Birjand Univ Med Sci..

[19] Azarfarin R, Seyedhejazi M, Golzari SE, Bilehjani E, Ghabili K, Alizadehasl A (2013). Do pediatric patients undergoing cardiac surgeries require larger-size cuffed endotracheal tubes? A prospective study.. Paediatr Anaesth..

[20] Walker PP, Rose E, Williams JG (2003). Upper airways abnormalities and tracheal problems in Morquio's disease.. Thorax..

[21] Braunlin EA, Harmatz PR, Scarpa M, Furlanetto B, Kampmann C, Loehr JP (2011). Cardiac disease in patients with mucopolysaccharidosis: presentation, diagnosis and management.. J Inherit Metab Dis..

[22] Sugiyama T, Okutani R (2012). Difficult tracheal intubation in a child with Cornelia de Lange syndrome using a paediatric Intlock installed in a Pentax Airway Scope.. Anaesthesia..

[23] Torres MD, Calvo E, Fernandez Espla F, Gilsanz F (2010). Anesthetic management of an adult patient with Cornelia de Lange Syndrome.. Minerva Anestesiol..

